# Acute Unilateral Audiovestibulopathy due to Embolic Labyrinthine Infarction

**DOI:** 10.3389/fneur.2018.00311

**Published:** 2018-05-02

**Authors:** Zhong Liqun, Kee-Hong Park, Hyo-Jung Kim, Sun-Uk Lee, Jeong-Yoon Choi, Ji-Soo Kim

**Affiliations:** ^1^Department of Neurology, Dongzhimen Hospital Affiliated to Beijing University of Chinese Medicine, Beijing, China; ^2^Department of Neurology, Gyeongsang National University Hospital, Jinju, South Korea; ^3^Research Administration Team, Seoul National University Bundang Hospital, Seongnam, South Korea; ^4^Department of Neurology, Seoul National University College Medicine, Seoul National University Bundang Hospital, Seongnam, South Korea

**Keywords:** vertigo, hearing loss, inner ear, infarction, embolism

## Abstract

**Introduction:**

Labyrinthine infarction is a cause of acute audiovestibulopathy, but can be diagnosed only in association with other infarctions involving the brainstem or cerebellar areas supplied by the anterior inferior cerebellar artery (AICA) since current imaging techniques cannot visualize an infarction confined to the labyrinth. This case series aimed to establish embolic labyrinthine infarction as a mechanism of isolated acute audiovestibulopathy.

**Methods:**

We analyzed clinical features, imaging findings, and mechanisms of embolism in 10 patients (8 men, age range: 38–76) who had developed acute audiovestibulopathy in association with an obvious source of embolism and concurrent acute embolic infarctions in the non-anterior inferior cerebellar artery territories. The presence of audiovestibulopathy was defined when bedside or laboratory evaluation documented unilateral vestibular (head-impulse tests or caloric tests) or auditory loss (audiometry).

**Results:**

Six patients showed combined audiovestibulopathy while three had isolated vestibulopathy. One patient presented isolated hearing loss. Audiovestibular findings were the only abnormalities observed in nine patients. In all patients, MRIs documented single or multiple infarctions in the cerebellum (*n* = 5) or cerebral hemispheres (*n* = 5). Especially three patients showed single or scattered foci of tiny acute infarctions only in the cerebral hemispheres. Cardiac sources of embolism were found in eight, and artery-to-artery embolism was presumed in two patients.

**Conclusion:**

Selective embolism to the labyrinth may be considered in patients with acute unilateral audiovestibulopathy and concurrent acute infarctions in the non-AICA territories.

## Introduction

Even though acute vertigo or hearing loss may occur due to an infarction involving the labyrinth (1), current imaging techniques do not readily allow identification of isolated labyrinthine infarctions as a cause of acute audiovestibulopathy (2). Since the labyrinth is supplied by the internal auditory artery (IAA) that mostly stems from the anterior inferior cerebellar artery (AICA) (3, 4), labyrinthine infarction is usually accompanied by infarctions involving the brainstem and cerebellar structures supplied by the AICA (5). Thus, labyrinthine infarction has mostly been presumed when patients show a combined loss of hearing and peripheral vestibular function in association with brainstem or cerebellar infarctions in the territory of AICA (5). Otherwise, isolated labyrinthine infarction or ischemia has been described as an initial presentation of AICA infarctions based on subsequent progression of acute audiovestibulopathy in isolation into a full AICA territory infarction involving the brainstem and cerebellum (6, 7). Rarely, sudden deafness with or without caloric canal paresis may occur in association with infarctions involving the cerebellum or brainstem of non-anterior inferior cerebellar artery (non-AICA) territory, and the sudden deafness in these cases were explained by a dominant posterior inferior cerebellar artery (PICA) supplying the labyrinth or a relative ischemic vulnerability of the labyrinth (8). The senior author has experienced occasional consultations regarding the patients who developed acute vertigo or hearing loss in association with the imaging findings of acute embolic infarctions in the distant non-AICA territories. The referring doctors mostly wonder how those infarctions remote from the labyrinth in the non-AICA territories could cause vertigo or hearing loss. However, the most probable mechanism would be a separate embolism to the labyrinth, which is not visualized with current imaging techniques.

Herein, we report 10 patients with acute audiovestibulopathy due to embolic labyrinthine infarction that was diagnosed based on the presence of cardiac or artery-to-artery sources of embolism and concurrent embolic infarctions observed in the non-AICA territories. The purpose of this report is to propose selective embolism to the labyrinth as a mechanism of acute audiovestibulopathy. This report would extend the mechanisms of isolated audiovestibulopathy into embolic labyrinthine infarction, and aid in accurate identification and management of this rare but important disorder.

## Subjects and Methods

### Subjects

**Figure 1:**
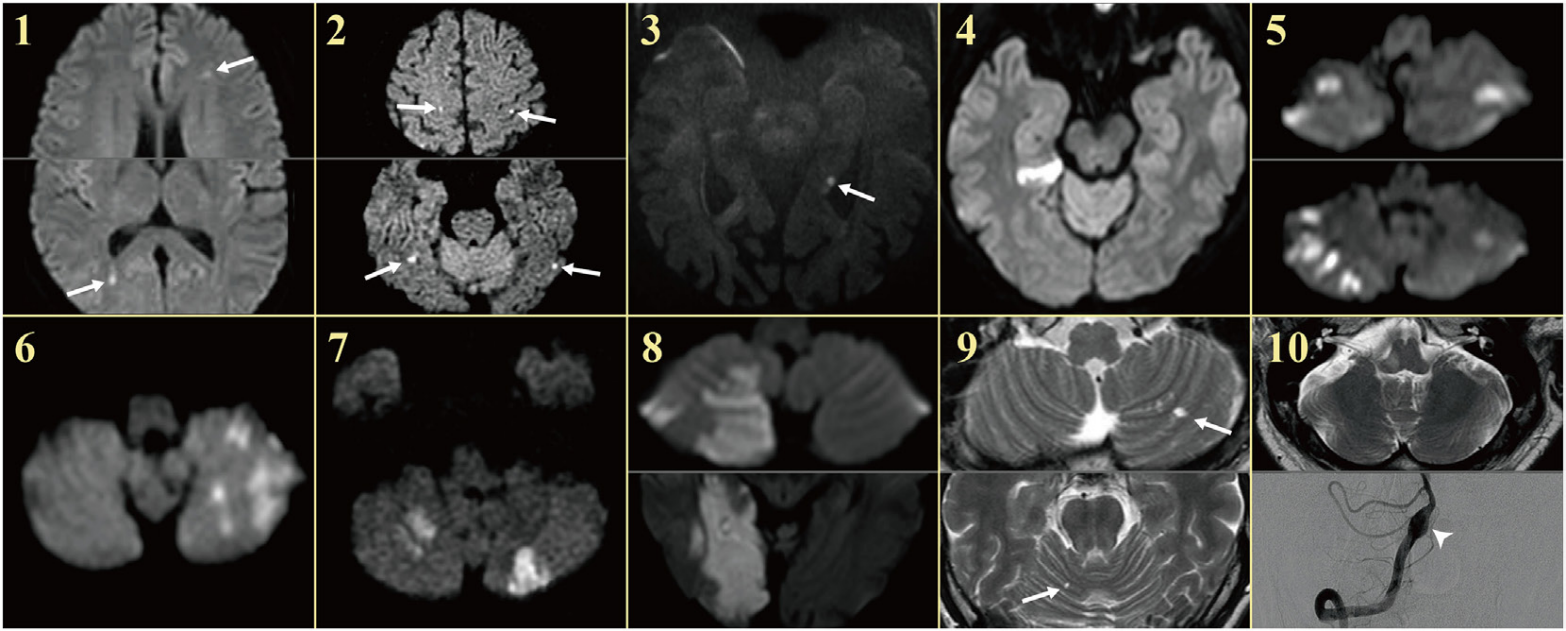
MRIs of the patients. Patients showed single or multiple acute infarctions in the cerebral hemispheres (arrows) or cerebellum concurrent with acute audiovestibulopathy.

From the 184 patients who had a diagnosis of AICA infarction at Seoul National University Bundang Hospital from 2003 to 2017, we had recruited 10 patients (8 men, age range: 38–76) who developed acute audiovestibulopathy in association with an obvious source of embolism and concurrent acute embolic infarctions in the non-AICA territories, which was demonstrated on MRIs (Figure 1). The presence of audiovestibulopathy was defined only when the patients developed vertigo or hearing loss, and bedside or laboratory evaluation documented unilateral vestibular [head-impulse tests (HITs) or caloric tests] or auditory loss (audiometry).

All the patients except one (patient 8) had MR imaging including diffusion-weight images and MR angiography within 7 days of symptom onset, but detailed audiovestibular evaluation became available 20 days to 5 months later in four patients (patients 1, 5, 7, and 9). One patient (patient 10) with acute unilateral audiovestibulopathy due to an embolism from the dissected vertebral artery (VA) was reported previously (9).

All experiments followed the tenets of the Declaration of Helsinki. This study was approved by the Institutional Review Board of Seoul National University Bundang Hospital (B-1711-432-111).

### Evaluation of Nystagmus

After bedside evaluation of spontaneous (SN) and gaze-evoked nystagmus (GEN) during visual fixation, and SN and head-shaking nystagmus (HSN) without visual fixation in darkness using video-Frenzel goggles (SLMED, Seoul, Korea) (10), nystagmus was also recorded binocularly using a video-oculography (SMI, Teltow, Germany) at a sampling rate of 60 Hz (11). While wearing the video-oculography goggles in the seated position, SN was recorded both with and without visual fixation during straight-ahead gaze. GEN was recorded in the horizontal (±30°) and vertical (±20°) planes. HSN was induced by a passive head-shaking. The patients’ head was grasped firmly with both hands, and shaken horizontally in a sinusoidal fashion at a rate of 2.8 Hz with an approximate amplitude of ±10° for 15 s (11). To induce positional nystagmus, the patients lay supine from sitting and turned their head to either side while supine. Then the patients were moved from the supine to sitting positions and the head was bent forward (12). The patients were also subjected to straight head hanging, and Dix–Hallpike maneuver to either side (13). Presence of positional nystagmus was defined as described previously (14).

### Head-Impulse Tests

Bedside HITs were performed manually with a rapid rotation of the head of ~20° amplitude in the planes of the horizontal and vertical canals. HITs were considered abnormal if a corrective saccade repetitively supplemented the inadequate slow phase in the plane of the semicircular canal stimulated (15). HITs were also quantified for all six semicircular canals using a magnetic search coil technique (Skalar, Delft, The Netherlands) (16, 17), or video-based equipment (SLVNG, SLMED, South Korea) (18). The normal ranges of video HIT gains were 0.88–1.27 for the horizontal canal, 0.75–1.25 for the anterior canal, and 0.77–1.13 for the posterior canal.

### Bithermal Caloric Tests

The caloric stimuli comprised alternate periods of irrigation for 25 s with 150–250 mL of cold (30°C) and hot (44°C) water. Nystagmus was recorded binocularly with video-oculography (ICS Medical, Schaumburg, IL, USA). Asymmetry of the vestibular function was calculated using Jongkees’ formula, and caloric paresis was defined as a response difference of at least 25% between the ears (11).

### Other Neurotologic Evaluation

Patients also underwent measurements of ocular torsion and subjective visual vertical (SVV), and cervical and ocular vestibular-evoked myogenic potentials (VEMPs). Detailed methods of each test have been described previously (19–22).

### MRI

All patients had MRI whose protocol included diffusion (DWI)-, T1-, and T2-weighted gradient-echo axial imaging, T1-weighted sagittal imaging using a 3.0- or 1.5-T unit (Intera; Philips Medical Systems, Best, The Netherlands). The imaging parameters were 4,800/100 [repetition time (ms)/echo time (ms)] for T2-weighted imaging, 500/11 for T1-weighted imaging, and 700/23 for gradient-echo imaging with a section thickness of 3 or 5 mm, a matrix size of 256 × 256 (interpolated to 512 × 512), and a field-of-view of 200–220 mm. DWIs were obtained using the following parameters; *b* = 1,000, 4,119/89 (repetition time/echo time), a section thickness of 3 or 5 mm, a matrix of 128 × 128 (interpolated to 256 × 256), and a field-of-view of 220 mm. For the patients with normal MRIs initially, follow-up MRIs were arranged within 7 days of symptom onset. In addition, all the patients had MR angiography of the intracranial vessels. The vascular territories were determined according to previously validated MR-anatomical templates (23, 24).

### Representative Case

#### Patient 1

**Figure 2:**
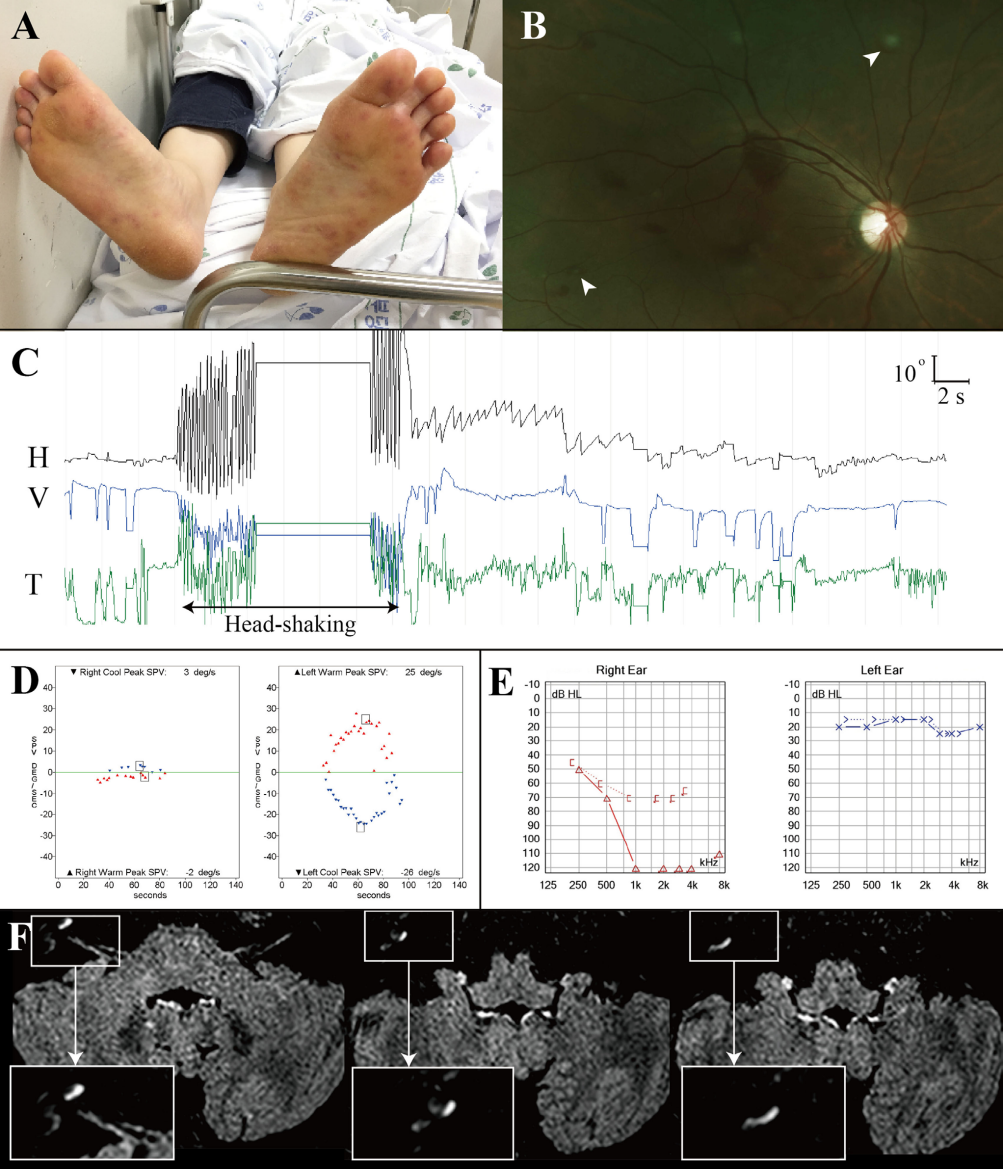
Combined audiovestibulopathy due to cardiac septic embolism. This 42-year-old woman (patient 1) with right audiovestibulopathy from cardiac septic embolism shows tender maculopapular rashes in both feet **(A)**, multiple scattered retinal hemorrhages with a white center (Roth spots, arrow heads) in both eyes **(B)**, left beating nystagmus after horizontal head-shaking [**(C)**, H = horizontal position of the left eye, V = vertical position of the left eye, T = torsional position of the left eye. In each recording, upward deflection indicates rightward, upward, and clockwise eye motion.] Patient 1 showed right caloric paresis of 81% [**(D)**, SPV = slow phase velocity], complete hearing loss in the right ear **(E)**, and gadolinium enhancements of the vestibule, cochlea, and semicircular canals [insets, **(F)**].

A previously healthy 42-year-old woman with fever, headache and sore throat for three days developed sudden bilateral visual loss and dysarthria, and lapsed into confusion and severe irritability that required sedation. Examination at that time showed tachycardia (142 beats/minute), body temperature of 39.6°C, neck stiffness, and tender maculopapular rashes in both hands and feet (Janeway lesion, Figure 2A). Fundus examination disclosed multiple scattered retinal hemorrhages with a white center (Roth spots) in both eyes (Figure 2B). Hematologic assays showed leukocytosis of 12,700/μL (neutrophil 94%), hemoglobin of 9.6 g/dL (hematocrit at 29.9%), and platelet counts at 135,000/μL. CSF study disclosed pleocytosis (1,020/mm^3^, neutrophil 80%), RBC of 200/mm^3^, protein at 166.0 mg/dL, and glucose of 55 mg/dL (serum glucose at 122 mg/dL). Under the suspicion of bacterial meningitis, the patient was placed on 2.6 g of vancomycin and 4 g of ceftriaxone per day. A methicillin-resistant *Staphylococcus aureus* was identified on blood culture later. Subsequent transthoracic echocardiography revealed a hypermobile vegetation (1.7 cm × 1.1 cm) attached to the mitral valve, which led to an operation for a valve replacement 20 days after the symptom onset.

On regaining consciousness 1 day after the operation, she reported dizziness, and hearing loss and tinnitus in the right ear. Examination showed no SN nystagmus either with or without visual fixation, but horizontal head-shaking induced left beating nystagmus (Figure 2C). Horizontal saccades and smooth pursuit were normal. She tended to fall to the right on attempted standing or walking. Other neurologic examination was unrevealing without weakness, sensory changes, or cerebellar dysfunction. Video HITs were abnormal for the right horizontal and posterior canals. Bithermal caloric tests showed right canal paresis of 81% (Figure 2D). Pure-tone audiometry documented right hearing loss (Figure 2E). SVV was normal. Cervical and ocular VEMPs showed no responses during right ear stimulation. Brain MRIs disclosed gadolinium enhancements of the vestibule, cochlea, and semicircular canals on the right side (Figure 2F) as well as multiple scattered infarctions in the frontal and occipital lobes (Figure 1, 1). The findings of MR angiography were normal. These findings were consistent with multiple infarctions due to septic embolism from cardiac vegetation. After 6 weeks of IV vancomycin and anticoagulation, she was discharged with minimal dizziness left although the tinnitus and hearing loss remained unchanged.

#### Patient 3

A 73-year-old man with a history of adrenal insufficiency, operations for colon cancer, patent foramen ovale, and pericardiac effusion was referred for further evaluation of acute vertigo and acute infarction observed on MRIs. He had developed acute vertigo and nausea/vomiting on the way to the bathroom just after awakening in the morning 6 days before the referral. He denied associated tinnitus, aural fullness, hearing loos, or sensorimotor symptoms. The vertigo had lasted into the afternoon with severe imbalance, and subsided after symptomatic medication with a diagnosis of vestibular neuritis at a local hospital. He was then referred to our ENT department where the MRI and MR angiography were arranged, and acute infarction was identified.

**Figure 3:**
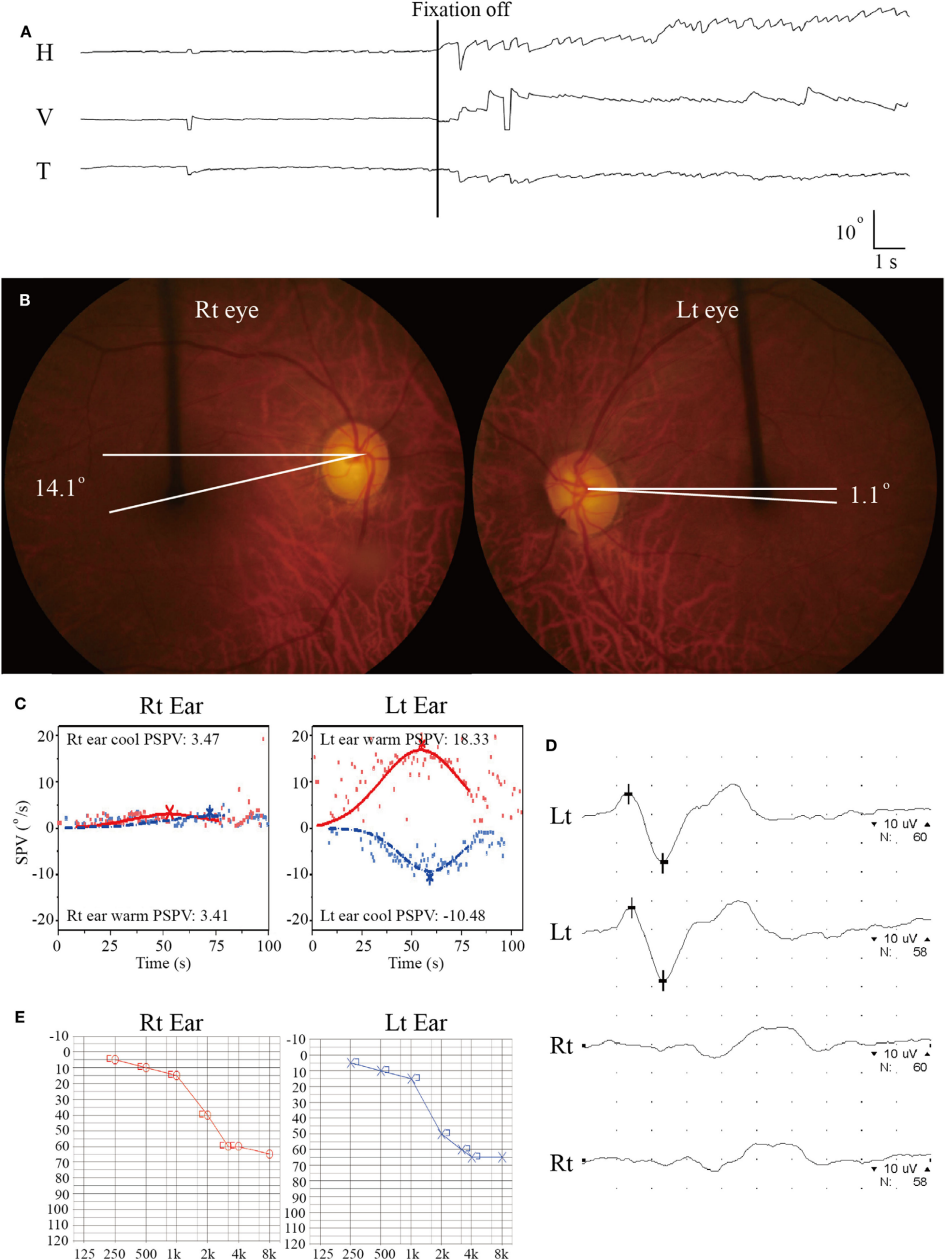
Isolated vestibulopathy in patient 3 with patent foramen ovale. **(A)** Video-oculography shows spontaneous nystagmus beating leftward, upward, and counterclockwise without fixation. H = horizontal position of the left eye, V = vertical position of the left eye, T = torsional position of the left eye. In each recording, upward deflection indicates rightward, upward, and clockwise eye motion. **(B)** Rightward ocular torsion is observed on fundus photos (normal range = 0–12.6^O^, positive values indicate an extorsion). **(C)** Bithermal caloric tests documented complete right canal paralysis. SPV = slow phase velocity, PSPV = peak SPV. **(D)** Ocular vestibular-evoked myogenic potential show no wave formation during right ear stimulation. **(E)** Pure-tone audiometry shows symmetric responses between the ears (Lt = left, Rt = right).

On evaluation 6 days after symptom onset, he showed SN nystagmus beating leftward, upward, and counterclockwise without fixation (Figure 3A). Bedside HITs were positive for the right horizontal canal. Rightward ocular torsion was observed on fundus photos (Figure 3B). SVV was tilted to the right either monocular [13.9 with right eye (normal range = −3.1 to 3.0), 16.0 with left eye (normal range = −3.8 to 3.1), positive values indicate a rightward tilt] or binocular viewing (13.9, normal range = −2.4 to 2.6). Bithermal caloric tests showed complete right canal paralysis (Figure 3C). Ocular VEMPs showed no responses during right ear stimulation (Figure 3D), but findings of cervical VEMPs were symmetric. Audiometry was normal (Figure 3E). Brain MRIs disclosed a tiny acute infarction in the tail portion of left hippocampus (Figure 1, 3). The findings of MR angiography were normal.

## Results

### Clinical Features

Of the 10 patients, 7 presented both vestibular (nausea/vomiting, dizziness/vertigo, or unsteadiness) and auditory (tinnitus or hearing loss) symptoms while three (patients 3, 4, and 8) had only vestibular symptoms (Table 1). Bedside hearing evaluation using a tuning fork showed sensorineural type hearing loss on the side reported by the patients. Except the one (patient 10) with concurrent lateral medullary infarction (LMI) due to VA dissection, audiovestibular findings were the only abnormalities observed. All patients showed a unilateral damage to the vestibular (*n* = 9) or cochlear (*n* = 7) labyrinth, which was documented with HIT, caloric tests or audiometry. In a patient (patient 7) with dizziness, tinnitus, and hearing loss at presentation, only right hearing loss was documented on audiometry without abnormal HIT or caloric paresis (isolated hearing loss). Except three, two with vestibular evaluation 20 days (patient 1) and 5 months (patient 7) after the event, and the other (patient 8) with a rapid improvement, all seven patients showed SN nystagmus that was invariably beat away from the damaged ear or the ear with caloric paresis (patient 6). Detailed findings of additional neurotological evaluation are provided in the Table S1 in Supplementary Material.

**Table 1:** Clinical findings of the patients.

Pt	Age/sex	Embolic source	Vestibular Sx	Auditory Sx	HIT	Caloric paresis	Hearing loss (pure-tone average^a^)	Onset-to-MRIs	Concomitant infarctions
1	F/42	Cardiac septic vegetation	Vertigo	Tinnitus/hearing loss, right	Right	Right (81%)	Right (18 dB)	Within 5 days^b^	Inferior frontal (MCA–ACA borderzone, left) and occipital (PCA, right) periventricular white matter
2	M/68	Coronary angioplasty	Vertigo/nausea	Hearing loss, left	Left	Left (46%)	Left (69 dB)	2 h	Precentral gyrus (ACA, right; MCA, left), occipitotemporal gyrus (PCA, bilateral)
3	M/73	PFO	Vertigo	–	Right	Right (100%)	Normal	6 days	Parahippocampal gyrus (PCA, left)
4	F/60	VA occlusion, right, V4 stenosis, left (>70%)	Vertigo	–	Right	Normal	Normal	5 h	Parahippocampal gyrus (PCA, right)
5	M/38	PFO	Vertigo/unsteadiness	Tinnitus/hearing loss, right	Normal^c^	Right (82%)	Right (63 dB)	2 days	Cerebellum (PICA, bilateral)
6	M/76	Atrial fibrillation	Dizziness/unsteadiness	Tinnitus/hearing loss, left	Normal	Right (84%)	Left (36 dB)	1 day	Cerebellum (PICA, left)
7	M/44	PFO	Dizziness	Tinnitus/hearing loss, right	Normal	None	Right (30 dB)	3 days	Cerebellum (PICA, bilateral)
8	M/75	Atrial fibrillation	Vertigo	–	Normal^c^	Right (63%)	Normal	7 h	Lingual, parahippocampal gyrus (PCA, right), cerebellum (PICA, right)
9	M/45	MS	Dizziness	Hearing loss, left	Left	Left (73%)	Left (68 dB)	4 months	Cerebellum (SCA, bilateral)
10	M/51	VA dissection without stenosis	Vertigo	Hearing loss, right	Right	Right (72%)	Right (15 dB)	7 h	LMI, right

*ACA, anterior cerebral artery; HIT, head-impulse tests; LMI, lateral medullary infarction; MCA, middle cerebral artery; MS, mitral stenosis; PFO, patent foramen ovale; PCA, posterior cerebral artery; PICA, posterior inferior cerebellar artery; Pt, patient; SCA, superior cerebellar artery; Sx, symptoms; VA, vertebral artery*.

*^a^Pure-tone average at 0.5 Hz, 1 kHz, 2 kHz, and 3 kHz (AMA guides, 6^th^ ed)*.

*^b^The exact onset time was unclear in this patient*.

*^c^Only bedside evaluation was performed in these patients with a dissociation in the results between HIT and caloric tests*.

### Imaging Findings and Mechanisms of Infarction

Except one (patient 1) with acute audiovestibulopathy from labyrinthine infarction due to cardiac septic emboli and gadolinium enhancements of the involved labyrinth (Figure 2F), all other patients showed no abnormalities in the labyrinth on MRIs. Instead, MRIs documented single or multiple infarctions in the cerebellum (*n* = 5) or cerebral hemispheres (*n* = 5, Figure 1). Especially three patients (patients 1, 2, and 3) showed single or scattered foci of tiny acute infarctions only in the cerebral hemispheres (Figure 1). Only one patient (patient 10) developed acute unilateral audiovestibulopathy in association with LMI due to VA dissection. MR angiography of the brain showed vascular abnormalities only in two patients, stenosis of left VA in one (patient 4) and dissection of right VA in the other (patient 10). Digital subtraction angiography was performed only in one patient (patient 10) in whom the inner ear was supplied by the PICA, but the vascular dominance could not be determined in the remaining patients. Follow-up MRIs were performed within 7 days of the first imaging in six patients, and found an additional infarction in only one (patient 6).

### Mechanism

Echocardiography was performed in eight (transesophageal in three, transthoracic in three, and both in two), and Holter monitoring in five patients. Cardiac sources of embolism were found in eight patients, patent foramen ovale in three, atrial fibrillation in two, valvular heart diseases in two (mitral stenosis in one, and aortic regurgitation in the other), and development of symptoms just after coronary angioplasty in the remaining one. Cardiac murmur was audible in two, and congestive heart failure was documented in another two. Artery-to-artery embolism was presumed in two patients, one with VA stenosis (patient 4) and the other with VA dissection (patient 10) without other identifiable stroke risk factors.

## Discussion

We report 10 patients with acute unilateral audiovestibulopathy in association with single or multiple foci of acute infarctions in the non-AICA territories. Given the location of infarctions confirmed on MRIs, we propose that concurrent embolism to the inner ear was the mechanism of acute unilateral audiovestibulopathy in these patients since current imaging techniques cannot readily disclose isolated labyrinthine infarctions. Our patients are unique in that the labyrinth was selectively affected by embolism among the structures irrigated by the AICA.

Acute deafness with or without caloric paresis may occur in infarctions involving non-AICA territories (8). However, those cases were reported only in association with infarctions involving the nearby brainstem or inferior cerebellum (8). In this study, 5 out of 10 patients developed audiovestibulopathy with concurrent embolic infarctions in the supratentorial areas. Only recently, sudden sensorineural hearing loss was described in a patient with patent foramen ovale (25). Even though combined vertigo and hearing loss is the most common presentation of labyrinthine infarction (7), the vestibular symptoms frequently take central stages and patients may not report hearing loss unless specifically inquired. Thus, evaluation of hearing should be a key component of the initial neurologic/vestibular examination in patients with acute vestibular syndrome.

**Figure 4:**
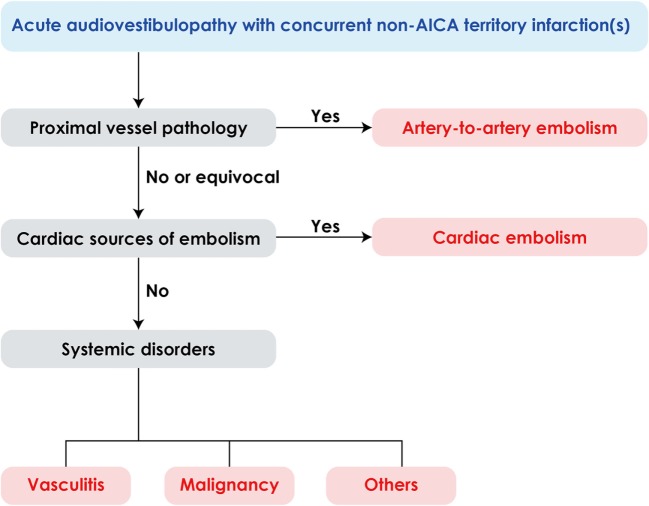
Algorithm for management of acute audiovestibulopathy and concurrent acute infarctions in the non-anterior inferior cerebellar artery (non-AICA) territories.

Since AICA infarction is mostly due to thrombotic occlusion of the artery at the take-off portion from the basilar artery, the predominant mechanism of labyrinthine infarction would be thrombosis especially when it is associated with infarctions involving the brainstem and anterior cerebellum supplied by the AICA (26–28). However, several cases of labyrinthine infarction have been ascribed to cardioembolism (5, 26, 28, 29) and isolated deafness have also been reported during embolization of a meningioma (30), bilateral knee replacement (31), or cardiopulmonary bypass surgery (32). Artery-to-artery embolism is another mechanism of labyrinthine infarction (9, 33). In addition, concurrent multiple small infarctions pose a possibility of systemic disorders, such as vasculitis, hypercoagulable state due to malignancy as well cardiac or artery-to-artery embolism (Figure 4) (34, 35). Thus, the neurotologists may be the first to detect serious systemic disorders likewise in our patient 1.

Previously, clinical features of labyrinthine infarction have mostly been described in patients with combined brainstem or cerebellar infarctions involving the AICA territory (5, 36), and only a few study reported isolated labyrinthine infarction due to an embolism (9) or as a limited form of AICA infarction before progression into a full AICA territory infarction involving the brainstem or cerebellum (6, 7). Our patients usually showed the symptoms and signs of both auditory and vestibular involvements. However, three patients developed dizziness/vertigo from unilateral peripheral vestibulopathy but without any evidence of auditory involvements while one patient had unilateral hearing loss without a vestibular involvement on caloric and HITs. The IAA, which usually originates from the AICA (4, 37), divides into two main branches within the internal auditory canal, the common cochlear and anterior vestibular arteries (37). The common cochlear artery further branches into the main cochlear and the vestibulocochlear arteries, the latter giving rise to the posterior vestibular artery and the cochlear ramus (37). The anterior vestibular artery irrigates the ampullae of the anterior and horizontal semicircular canals, the utricle and superior part of the saccule, while the posterior vestibular artery supplies the inferior part of the saccule and the ampulla of the posterior semicircular canal (37). Thus, even though the labyrinth is mostly involved in an all-or-none pattern in AICA infarction (5), each compartment of the labyrinth may be selectively involved when embolism involves a specific branch of the IAA, i.e., isolated vertigo or hearing loss. For example, isolated vertigo may occur without hearing loss when the anterior vestibular artery is only involved, and hearing loss may be an isolated finding when the common cochlear artery is selectively affected.

In our patients, the diagnosis of acute unilateral audiovestibulopathy from labyrinthine infarction was made based on concurrent embolic infarctions confirmed on MRIs, mostly in the distant vascular territories, and obvious source of embolism. Without these concomitant infarctions, the isolated unilateral audiovestibulopathy would have been ascribed to idiopathic or inflammatory disorders involving the labyrinth. Since those small ischemic foci can be easily missed even with MRIs and embolic sources could not be identified in some patients, we believe that the real incidence of acute audiovestibulopathy from embolic labyrinthine infarctions should have been higher than that we have estimated from our case series. Indeed, three of our patients showed only tiny ischemic foci on MRIs. Furthermore, the retrospective anecdotal case series limited systematic search for concurrent infarctions due to embolism or other causes in every patient with acute isolated vertigo or hearing loss. Thus, the proportion of patients with embolic labyrinthine infarction among our cohorts of AICA infarction may have under-represented the patients with this condition.

In conclusion, embolic labyrinthine infarction may be a cause of acute unilateral audiovestibulopathy. Since current imaging techniques cannot visualize isolated labyrinthine infarctions, the diagnosis of labyrinthine infarction could be inferred only from concurrent infarctions on MRIs in patients with acute audiovestibulopathy. Embolic sources should be sought in patients with acute audiovestibulopathy of unknown etiology.

## Ethics Statement

This study followed the tenets of the Declaration of Helsinki, and was performed according to the guidelines of Institutional Review Board of Seoul National University Bundang Hospital (B-1711-432-111). Waiver of informed consent was allowed by the Institutional Review Board for the retrospective chart review.

## Author Contributions

ZL analyzed and interpreted the data, and wrote the manuscript. K-HP, H-JK, S-UL, and J-YC conducted interpretation of the data and revision of the manuscript. J-SK conducted the design and conceptualization of the study, interpretation of the data, and revision of the manuscript.

## Conflict of Interest Statement

J-SK serves as an associate editor of Frontiers in Neuro-otology and on the editorial boards of the Journal of Clinical Neurology, Frontiers in Neuro-ophthalmology, Journal of Neuro-ophthalmology, Journal of Vestibular Research, Journal of Neurology, and Medicine. All other authors declare that the research was conducted in the absence of any commercial or financial relationships that could be construed as a potential conflict of interest.
