# Supplementary material for: Acute Unilateral Audiovestibulopathy due to Embolic Labyrinthine Infarction
Source: Front Neurol. 2018 May 2;9:311. doi: 10.3389/fneur.2018.00311 (PMC5940739; doi:10.3389/fneur.2018.00311)
Supplement: Supplementary file 1 [file table_1.doc]

Supplementary table. Neurotologic findings in the patients

| **Pt** | **Evaluation^*^** | **SN** | **HSN** | **VIN** | **GEN** | **OTR** | **SVV** | **oVEMPs** | **cVEMPs** |
| --- | --- | --- | --- | --- | --- | --- | --- | --- | --- |
| 1 | 20 days | - | Left |  | - | - | Normal | NR, right | NR, right |
| 2 | 3 days | R-CW | Right | Right | - | - | Left | ND | ND |
| 3 | 6 days | L-U-CCW | Left | Left | - | Right | Right | NR, right | Normal |
| 4 | 1 day | L | Left | Left | + | - | Normal | Normal | Normal |
| 5 | 2 months | L | Left | Left | - | - | Normal |  | NR, Right |
| 6 | 2 days | L | - | - | - | - | Left | ND | ND |
| 7 | 5 months | - | - | - | - | - | - | Normal | Normal |
| 8 | 1 day | - | - | - | - | - | Normal | ND | ND |
| 9 | 4 months | R | Right | Right | - | - | - | NR, left | NR, left |
| 10 | 1 day | L-CCW | ND | ND | + | - | Right | ND | ND |

cVEMPs=cervical vestibular-evoked myogenic potentials, CCW=counter-clockwise (from the patient’s perspective), CW=clockwise (from the patient’s perspective), GEN=gaze-evoked nystagmus, HSN=head shaking nystagmus, L=leftward, ND=not done, NR=no response, OTR=ocular tilt reaction, oVEMPs=ocular vestibular-evoked myogenic potentials, Pt=patients, R=rightward, SN=spontaneous nystagmus, SVV=subjective visual vertical, VIN=vibration-induced nystagmus

*The duration from the symptom onset to neurotological evaluation.
